# Premedication with Oral Alprazolam and Melatonin Combination: A Comparison with Either Alone—A Randomized Controlled Factorial Trial

**DOI:** 10.1155/2014/356964

**Published:** 2014-01-12

**Authors:** Krishna Pokharel, Mukesh Tripathi, Pramod Kumar Gupta, Balkrishna Bhattarai, Sindhu Khatiwada, Asish Subedi

**Affiliations:** ^1^Department of Anesthesiology and Critical Care, B. P. Koirala Institute of Health Sciences, Dharan 56700, Nepal; ^2^Department of Anesthesiology, Sanjay Gandhi Post Graduate Institute of Medical Sciences, Lucknow 226014, India; ^3^Department of Anesthesiology, Banaras Hindhu University, Varanasi 221005, India

## Abstract

We assessed if the addition of melatonin to alprazolam has superior premedication effects compared to either drug alone. A prospective, double blind placebo controlled trial randomly assigned 80 adult patients (ASA 1&2) with a Visual Analogue Score (VAS) for anxiety ≥3 to receive a tablet containing a combination of alprazolam 0.5 mg and melatonin 3 mg, alprazolam 0.5 mg, melatonin 3 mg, or placebo orally 90 min before a standard anesthetic. Primary end points were change in anxiety and sedation score at 15, 30, and 60 min after premedication, and number of patients with loss of memory for the five pictures shown at various time points when assessed after 24 h. One-way ANOVA, Friedman repeated measures analysis of variance, Kruskal Wallis and chi square tests were used as relevant. Combination drug produced the maximum reduction in anxiety VAS (3 (1.0–4.3)) from baseline at 60 min (*P* < 0.05). Sedation scores at various time points and number of patients not recognizing the picture shown at 60 min after premedication were comparable between combination drug and alprazolam alone. Addition of melatonin to alprazolam had superior anxiolysis compared with either drugs alone or placebo. Adding melatonin neither worsened sedation score nor the amnesic effect of alprazolam alone. This study was registered, approved, and released from ClinicalTrials.gov. Identifier number: NCT01486615.

## 1. Introduction

Benzodiazepines are amongst the most popular preoperative medication to produce anxiolysis, amnesia, and sedation for the patients coming for surgery [1]. Benzodiazepines are reported to be paradoxically associated with the increased episodes of arousal during sleep, restlessness, and hangover effects [2]. Alprazolam, a benzodiazepine class of antipsychotic drugs, is more anxioselective than the other premedicants of this group like midazolam, lorazepam, or diazepam [3]. It has also been reported to show arousal episodes [2].

Melatonin (N-acetyl-5-methoxytryptamine), an endogenous pineal hormone when given orally as premedicant, also results in preoperative anxiolysis and sedation [4, 5]. It neither impairs cognitive and psychomotor skills nor the quality of recovery [4, 5]. Its anxiolytic, sedative, hypnotic, analgesic, anti-inflammatory, antioxidative, and chronobiotic properties distinguish it as an attractive alternative premedicant [6].

Benzodiazepines are associated with the suppression of endogenous melatonin levels to probably show the increased episodes of arousal during sleep, restlessness, and hang over effects [2]. We hypothesize that the arousal episodes produced by alprazolam can be reduced by combining it with exogenous melatonin, hence might promote sound sleep to improve upon preoperative stress. To our knowledge, combination of melatonin and alprazolam as premedicant has not been evaluated. Hence, we designed this prospective, randomized, double blind placebo controlled factorial trial to assess whether addition of melatonin to alprazolam has any advantage over alprazolam or melatonin alone in premedication.

## 2. Methods

After getting approval from the institutional research ethics committee and written informed consent from each patient, we studied eighty ASA 1 and 2 patients, aged 18 to 65 yr having anxiety VAS score of three or more, planned for receiving general anesthesia for laparoscopic cholecystectomy. Patients taking analgesics, sedatives, antiepileptics, or antidepressants, suffering from obesity (BMI ≥ 28) or neuropsychiatric disease, having history of allergy to the study drugs were excluded.

With the help of computer generated random numbers that were concealed until after the consent was obtained, patients were assigned to one of the four groups of twenty each. Group one patients were administered a tablet containing the combination of oral alprazolam (0.5 mg) and melatonin (3 mg). Group two patients received alprazolam (0.5 mg). Group three patients received melatonin (3 mg). Group four patients were administered a similar looking placebo tablet. All patients received only one tablet with sips of plain water, approximately 90 min before surgery.

One day prior to surgery on the preanesthetic visit, all the patients were explained about the nature of the study and the various scales to be used. A 10 cm linear Visual Analogue Scale (VAS) [7] was used to assess their anxiety levels. The extremes of the VAS anxiety scale were marked as “no anxiety” at the 0 end and “anxiety as bad as ever can be” at the 10 cm end. Sedation was assessed on a five-point scale (0 = alert, 1 = arouses to voice, 2 = arouses with gentle tactile stimulation, 3 = arouses with vigorous tactile stimulation, and 4 = lack of responsiveness) [8] and orientation, with a three-point scale (0 = none, 1 = orientation in either time or place, and 2 = orientation in both) [4]. To test for the memory, recall of five different simple pictures and two events were assessed. Pictures to be used were sequentially numbered on the back and their names printed on the front.

Approximately 90 min prior to surgery, each patient was taken to a quiet room. Noninvasive blood pressure, heart rate, respiratory rate, and SpO_2_ were monitored. Then picture one (cup on a plate) and two (fruits) were shown 10 min before and just prior to the drug administration, respectively. Patients were asked to take the study medication orally with 15 mL of plain water according to the group assignment by an investigator not involved in the patient management or data collection thereafter. Anxiety, sedation, and orientation scores were assessed just prior to and at 15 min, 30 min, and 60 min after the drug administration. At these time points pictures three (birds), four (hare), and five (car) were also shown, respectively.

In the operating room, intravenous access was secured and meperidine 1 mg/kg administered intravenously. Then intravenous lidocaine 20 mg bolus was administered followed by propofol via infusion pump at 100 mL/h till loss of response to verbal command and loss of eyelash reflex were noticed. Vecuronium 0.1 mg/kg and isoflurane in oxygen were administered to maintain the adequate depth of anesthesia. After intubation, ventilation was adjusted to maintain normocapnia. Incremental doses of the injection meperidine (0.1 mg/kg) and vecuronium (0.02 mg/kg) were given as required. Intravenous diclofenac sodium 75 mg was administered slowly 15 min prior to completion of surgery for postoperative analgesia. After completion of surgery, intravenous neostigmine (50 *μ*gm/kg), and glycopyrrolate (10 *μ*gm/kg) were given to reverse the residual muscle paralysis. Anesthesia time (induction to emergence) was noted.

In the recovery room, patients received the standard postoperative care, including oxygen administration via face mask (6 L/min) and monitoring of heart rate, respiratory rate, noninvasive blood pressure and SpO_2_. We observed for any episodes of nausea, vomiting, dizziness, headache, and restlessness in the next 24 h. Intravenous ondansetron (4 mg) was given slowly to treat postoperative nausea and vomiting.

After 24 h following surgery, the patients were asked if they recalled the two events, viz. being transported to the operating room and intravenous cannula being inserted. They were also asked to have a free recall of the five pictures they were shown. Then, the first five pictures that were shown were mixed with five new pictures (of a horse, shoe, bicycle, elephant, and tiger) and they were asked to recognize the pictures which were shown before surgery. They were also asked whether they would like to receive the same premedication drug in the future if required.

The primary end points were change in anxiety, sedation, and orientation scores at various time points after premedication and the number of patients with loss of memory for the five pictures 24 h later. Secondary outcome was the number of patients who desired to receive the same premedication drug in the future if required.

### 2.1. Statistical Analysis

Before the study began, a sample size of 16 patients in each group was determined by a power analysis (*α*, 0.05; *β*, 0.10) on the basis of the assumption that there will be 50% reduction in anxiety VAS from baseline in the experimental groups and 4% in the placebo group. To compensate for drop out cases and shifting from normality in data distribution, 20 cases were studied in each group.

Data were tested for normal distribution using Kolmogorov-Smirnov test. To identify differences between groups, one-way analysis of variance (ANOVA) was used for normally distributed continuing data and chi square tests for categorical data. As the anxiety and sedation scores were not normally distributed; they were thus analyzed with nonparametric statistical methods. Friedman repeated measures analysis of variance followed by Wilcoxon tests with Bonferroni correction were used for within-group comparison of values between different time points. Kruskal Wallis tests with *post hoc *multiple comparisons by Mann Whitney *U* test were used for the comparison of values between the groups at each time points. Parametric data were expressed as the mean ± SD and nonparametric data as median (interquartile range). A *P* value < 0.05 was considered significant.

## 3. Results

**Figure 1:**
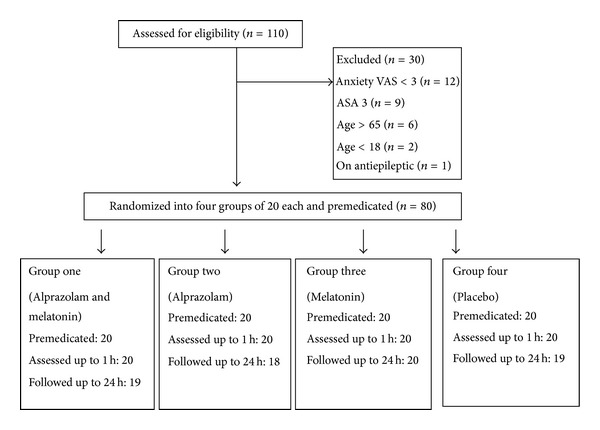
A flow diagram showing inclusion, exclusion, group allocation, intervention and follow up.

One hundred and ten patients were assessed for eligibility, 98 patients had anxiety VAS ≥ 3. Among the 80 enrolled patients who were observed till two hours after premedication, the surgery was postponed in five patients because of limited operating room time. These patients were discharged on the same day and were not available for the assessment at 24 h after premedication (Figure 1). Patients in the four groups were comparable in demographic characteristics and perioperative parameters (Table 1).

### 3.1. Report on Anxiety

**Figure 2:**
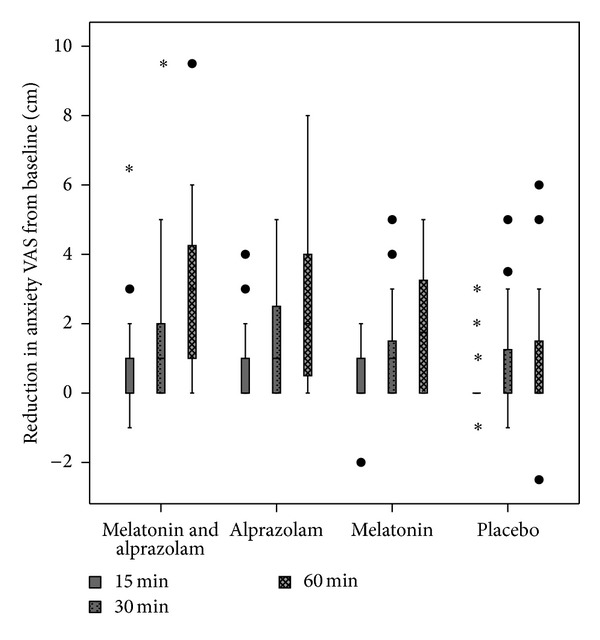
Change in anxiety VAS (median (IR)) relative to baseline after premedication. ∙ = outlier, ∗ = extreme value.

VAS anxiety scores were significantly (*P* < 0.001) reduced relative to baseline at various time points in all the groups {*χ* ^2^(df) = 43.6(3) for melatonin and alprazolam, *χ* ^2^(df) = 39.5(3) for alprazolam, *χ* ^2^(df) = 31.5(3) for melatonin} except in placebo (*χ* ^2^(df) = 6.8, *P* > 0.05) (Figure 2). Wilcoxon tests with Bonferroni correction (effects reported at a 0.0167 level of significance) revealed that these within group differences were significant only at 30 min and 60 min relative to baseline in all the three groups.

When compared in between the groups, the reduction in anxiety VAS from baseline were significantly different only at 60 min after premedication {*H*(3) = 11.8,  *P* = 0.008}. Mann Whitney tests with Bonferroni correction (effects reported at a 0.01 level of significance) were used to follow up this finding. The reduction in anxiety VAS {median (interquartile range)} was significantly different only between the groups receiving the combination drugs {3(1.0–4.3)cm} and placebo {0(0–1.7) cm}  (*U* = 86.5, *z* = −3.18,  *P* = 0.008) (Figure 2).

### 3.2. Report on Sedation

Sedation scores were significantly increased relative to baseline at various time points in all the groups {*χ* ^2^(df) = 34.9(3), *P* < 0.001 for melatonin and alprazolam combination, *χ* ^2^(df) = 31.9(3), *P* < 0.001 for alprazolam, *χ* ^2^(df) = 18.4(3), *P* < 0.001 for melatonin} including placebo (*χ* ^2^(df) = 12.6(3), *P* = 0.006) (Figure 2). To follow up this finding, Wilcoxon tests with Bonferroni correction were applied; so all effects are reported at a 0.0167 level of significance. While the increment in sedation score relative to baseline was significant at only 60 min in placebo, the differences were significant at both 30 and 60 min after premedication in rest of the three groups.

When the sedation scores {median (interquartile range)} were compared in between groups, the difference was significant at only 60 min after premedication {*H*(3) = 18.5, *P* < 0.001}. Mann Whitney tests with Bonferroni correction (effects reported at a 0.00714 level of significance) were used to follow up this finding. The difference was significant only between the groups receiving combination drugs {1 (1.0–1.75)} and placebo {0 (0-1)} (*U* = 86.5, *z* = −3.18,  *P* = 0.008), and between the groups receiving the combination drugs {1 (1.0–1.75)} and melatonin {0.5 (0-1)}  (*U* = 85, *z* = −3.33, *P* = 0.001). Details of the sedation scores are given in Table 2.

### 3.3. Report on Orientation

All the patients had orientation score of 2 at 15 min, 30 min and 60 min after premedication.

### 3.4. Report on Amnesia

More number of patents in groups receiving the combination drugs and alprazolam (9 each) did not recognize the picture shown at 60 min after premedication compared to groups receiving melatonin and placebo (2 each) (*P* < 0.05). Amnesia for two events was notable in maximum number of patients in the group receiving the combination of alprazolam and melatonin. However, the difference was statistically significant only between the groups receiving the combination drugs (5 (26%)) and placebo (0) for only one event (shifting to the operating room) (Table 3).

### 3.5. Patient's Perception

Except for the one patient who was not sure, all the patients who received the combination of alprazolam and melatonin stated they would prefer to have the same premedication in the future. Two (11%) in alprazolam group, five (25%) in melatonin group and six (33%) patients in placebo group expressed a change to other premedicant in future.

### 3.6. Safety Profile

There was no statistical difference between the groups in the number of people reporting occurrence of nausea, vomiting, dizziness, headache, or restlessness (Table 1).

## 4. Discussion

We have found that the melatonin alprazolam combination reduced anxiety level to a greater extent than the either drug alone. All three premedicants put the patients to sleep earlier than placebo. The combined premedication did not worsen sedation than the alprazolam. Amnesia was notable in patients receiving melatonin alprazolam combination and alprazolam alone. Almost all the patients receiving the melatonin alprazolam combination preferred to have the same premedicant in the future. All the three premedication drugs were safe in terms of the side effects observed till 24 h after surgery.

Benzodiazepines are the most common premedicant before surgery to alleviate preoperative anxiety [1]. Benzodiazepines enhance the effect of the neurotransmitter gamma amino butyric acid (GABA), to show sedation, anxiolysis, amnesia, muscle relaxation, and anticonvulsant effects. In a Belgian study, oral alprazolam (0.5 mg) produced significant levels of sedation (sedation VAS 23 ± 20 mm) compared to placebo (sedation VAS 7 ± 20 mm) at 60 to 90 min after premedication in patients planned for day care surgery [8]. In our patients alprazolam produced more sedation scores than placebo at 60 min after premedication, but the difference was not statistically significant. However, our patients who received alprazolam got sedated half an hour earlier than placebo.

Melatonin is a naturally occurring pineal hormone in the human body which regulates the circadian rhythm [9]. Its levels are high during night time sleep [9]. Oral administration of 1–5 mg of melatonin results in plasma levels of 10–100 times more than the observed endogenous night time levels [9]. Oral melatonin (5 mg) is reported to cause significant sedation at 60–90 min after premedication [4]. We too found that the melatonin administration was associated with earlier onset of sleep than the placebo.

Interestingly, all our patients got significantly sedated at one hour of the stay in preoperative waiting room from their baseline level of sedation score. It could be corroborated with their stay in isolation at the quiet preoperative waiting room where they were shifted for the study purpose.

We have found that adding melatonin to alprazolam reduced anxiety levels more than either of the two drugs given alone. Although the exact mechanism of the action of melatonin is still not known, there is accumulating evidence that a synergy exists between the melatonergic and GABAergic systems. It has been reported that the melatonin administration is associated with significant, dose-dependent increases in GABA concentrations in the central nervous system [10]. We observed that the added dose of melatonin in combination with alprazolam caused similar levels of sedation as seen with alprazolam alone suggesting that the melatonin in doses we used did not worsen sedation levels.

Benzodiazepines are reported to impair psychomotor performance [5, 8]. The reports of the effect of melatonin on the orientation score with respect to time and place has varied in the literature [4, 5, 11]. Since we only recorded the orientation score to time and place in our patients, it remained preserved in perioperative period of study.

Several studies have reported that melatonin lacks antegrade amnesia [4, 5, 11, 12]. The incidence of amnesia in our patients receiving melatonin was low, and comparable to placebo. Alprazolam at doses of 0.5 mg and higher has been reported to impair immediate and delayed recall and recognition [13]. Adding melatonin to alprazolam did not change the amnesic effects in our patients.

Interestingly majority of our patients premedicated with the combination of melatonin with alprazolam, expressed their desire to get similar premedication in future too. Benzodiazepines are associated with episodes of arousal during sleep [2] and later also show hangover effects [4] as they decrease the duration of rapid eye movement and slow wave sleep [14]. In contrast, melatonin is known to improve the quality of sleep [15]. While benzodiazepines are reported to decrease melatonin levels [2], the combined medication probably improved the quality of sleep in preoperative period to improve liking of the patients for the combined premedication than either drug alone or placebo. However, as our study was not designed to address the precise reason for preferring for the combined medication, for example, better anxiolysis or sleep or both, we cannot comment on this further.

All the drugs we used were safe in terms of adverse effects encountered. The minor problems that our patients complained were headache, restlessness, dizziness, nausea, and vomiting and their frequencies were similar in patients of all study groups. However, our study was not statistically powered to detect the incidence of adverse events. Both melatonin and alprazolam are reported to be safe [16, 17] and serious untoward effects have not been documented in the therapeutic range so far.

The limitations of our study include a small sample size considering the factorial trial of the two premedicant drugs and limited tests of the orientation score and the delayed visual episodic memory in place of the detailed psychomotor and battery of memory tests. Further, we also could not measure the blood levels of the studied drugs because of lack of facility.

## 5. Conclusion

While melatonin alprazolam combination reduced anxiety better than either drug alone, it produced sedation and amnesia to a similar degree as alprazolam alone. More patients desired to receive the melatonin and alprazolam combination as premedicant in the future. Authors recommend the use of combination of alprazolam with melatonin orally as premedicant in surgical patients.

## Figures and Tables

**Table 1:** Patient data and perioperative variables. Values are expressed as number (%), mean ± SD or median (interquartile range) as applicable. No significant differences.

	Melatonin and Alprazolam	Alprazolam	Melatonin	Placebo
Patients data				
Number of patients (*n*)	20	20	20	20
Gender (male/female)	6/14	4/16	4/16	5/15
Age (yr)	38 ± 14	37 ± 10	34 ± 11	36 ± 13
Weight (kg)	52 ± 7	52 ± 7	57 ± 11	57 ± 12
History of previous surgery (*n*)	11 (55)	8 (40)	8 (40)	9 (45)
VAS baseline (cm)	5 (3.6–6)	5 (4.2–7)	5 (4–6)	5 (4–6)
Perioperative variables				
Number of patients (*n*)	19	18	20	18
Propofol (mg) consumed until				
Loss of verbal command	66 ± 21	59 ± 21	79.2 ± 4	75.8 ± 25
Loss of eyelash reflex	73 ± 22	62 ± 22	79 ± 35	80.8 ± 29
Anesthesia time (min)	77 ± 34	73 ± 21	66 ± 18	72 ± 18
Patients (*n*) with				
Nausea and vomiting	8 (42)	6 (33)	7 (35)	9 (50)
Headache	4 (21)	3 (17)	4 (20)	5 (28)
Dizziness	3 (16)	2 (11)	5 (25)	4 (22)
Restlessness	7 (37)	9 (50)	6 (30)	8 (44)

**Table 2:** Showing sedation in the four groups at various time points after premedication. Values are expressed as number of patients (%).

	Melatonin and Alprazolam (*n* = 20)	Alprazolam (*n* = 20)	Melatonin (*n* = 20)	Placebo (*n* = 20)
Baseline				
Alert	18 (90)	20 (100)	17 (85)	20 (100)
Arouses to voice	2 (10)	0 (0)	3 (15)	0 (0)
15 min after premedication				
Alert	13 (65)	16 (80)	13 (65)	18 (90)
Arouses to voice	4 (20)	4 (20)	6 (30)	2 (10)
Arouses with gentle tactile stimulation	3 (15)	0 (0)	1 (5)	0 (0)
30 min after premedication				
Alert	7 (35)	10 (50)	9 (45)	15 (75)
Arouses to voice	8 (40)	6 (30)	8 (40)	4 (20)
Arouses with gentle tactile stimulation	4 (20)	4 (20)	3 (15)	1 (5)
Arouses with vigorous tactile stimulation	1 (5)	0 (0)	0 (0)	0 (0)
60 min after premedication				
Alert	3 (15)	6 (30)	10 (50)	12 (60)
Arouses to voice	8 (40)	9 (45)	7 (35)	8 (40)
Arouses with gentle tactile stimulation	4 (20)	4 (20)	3 (15)	0 (0)
Arouses with vigorous tactile stimulation	5 (25)	1 (5)	0 (0)	0 (0)

**Table 3:** Showing the result of assessment of memory for the five pictures shown at various time points related to premedication on the previous day and the two events. Values are expressed as number of patients (%).

	Melatonin and Alprazolam (*n* = 19)	Alprazolam (*n* = 18)	Melatonin (*n* = 20)	Placebo (*n* = 18)	*P* value
Picture 1 (shown 10 min prior to premedication)					
Recalled	15 (79)	17 ((94)	15 (75)	14 (78)	0.178
Recognized	19 (100)	18 (100)	20 (100)	18 (100)	
Picture 2 (shown just prior to premedication)					
Recalled	15 (79)	14 (78)	13 (65)	15 (83)	0.965
Recognized	19 (100)	18 (100)	20 (100)	18 (100)	
Picture 3 (shown 15 min after premedication)					
Recalled	15 (79)	17 (94)	13 (65)	13 (72)	0.051
Recognized	17 (89)	18 (100)	20 (100)	18 (100)	
Picture 4 (shown 30 min after premedication)					
Recalled	9 (47)	8 (44)	10 (50)	14 (78)	0.255
Recognized	16 (84)	15 (83)	19 (95)	18 (100)	
Picture 5 (shown 60 min after premedication)					
Recalled	4 (21)	5 (28)	13 (65)	14 (78)	0.003
Recognized	10 (53)	9 (50)	18 (90)	16 (89)	
Event 1 (transfer to operating room)					
Recalled	14 (74)	14 (78)	19 (95)	18 (100)	0.047
Event 2 (intravenous cannulation)					
Recalled	12 (63)	14 (78)	18 (90)	16 (89)	0.135
